# Caregiver parenting practices, dietary diversity knowledge, and association with early childhood development outcomes among children aged 18-29 months in Zanzibar, Tanzania: a cross-sectional survey

**DOI:** 10.1186/s12889-022-13009-y

**Published:** 2022-04-15

**Authors:** Allyson L. Russell, Elizabeth Hentschel, Isabel Fulcher, Matteo Santangelo Ravà, Gulam Abdulkarim, Omar Abdalla, Samira Said, Halima Khamis, Bethany Hedt-Gauthier, Kim Wilson

**Affiliations:** 1 D-tree International, Zanzibar, Tanzania; 2 Department of Global Health and Population, Harvard T.H. Chan School of Public Health, Boston, MA USA; 3 Department of Global Health and Social Medicine, Harvard Medical School, Boston, MA USA; 4 Ministry of Health, Revolutionary Government of Zanzibar, Zanzibar, Tanzania; 5 Department of Biostatistics, Harvard T.H. Chan School of Public Health, Boston, MA USA; 6 Boston Children’s Hospital, Boston, MA USA

**Keywords:** Child development, CREDI, Caregiver engagement, Early stimulating activities, Nurturing care, Home environment, Community health volunteer, Dietary diversity, Digital health, Tanzania, Zanzibar

## Abstract

**Background:**

Many children in low- and middle-income countries fail to reach their cognitive potential, with experiences before age 3 critical in shaping long-term development. Zanzibar’s Jamii ni Afya program is the first national, digitally enabled community health volunteer (CHV) program promoting early childhood development (ECD) following the Nurturing Care Framework within an integrated maternal and child healthcare package. Using program baseline data, we explored home environment, caregivers’ parenting, health and nutrition knowledge and practices, and ECD outcomes in Zanzibar.

**Methods:**

We conducted a national household survey among 499 children aged 18-29 months using two-stage cluster sampling in February 2019. The primary outcome was child development score measured using the Caregiver Reported Early Developmental Index (CREDI), with higher scores representing higher levels of child development. We analyzed CREDI scores, along with MICS questions on parenting knowledge, practices, and characteristics of the home environment. We developed multivariate regression models to assess associations between caregiver-child interactions, knowledge of dietary diversity, and ECD.

**Results:**

Ten percent of children had overall CREDI z-scores 2 standard deviations [SD] or more below the global reference population mean, with 28% of children at risk of developmental delay with z-scores 1 SD or more below the mean. Cognitive and language domains were of highest concern (10.2 and 12.7% with z-score < − 2 SD). In 3-day recall, 75% of children engaged in ≥4 early stimulating activities with all caregivers averaging 3 total hours of play. CREDI scores were positively associated with greater frequency of caregivers’ engagement (β = 0.036, *p* = 0.002, 95%CI = [0.014, 0.058]), and dietary diversity knowledge (β = 0.564, *p* < 0.001, 95%CI = [0.281, 0.846]).

**Conclusions:**

Our findings demonstrate a positive association between both the frequency of caregiver child interactions and knowledge of adequate dietary diversity, and ECD outcomes. This aligns with global evidence that promoting early stimulation, play and learning opportunities, and dietary diversity can improve developmental outcomes. Further study is needed to establish causal relationships and assess the impact of ECD programming in Zanzibar.

**Supplementary Information:**

The online version contains supplementary material available at 10.1186/s12889-022-13009-y.

## Key message

One in ten children aged 2 years in Zanzibar exhibit significant concerns for developmental delay. Our research shows that the frequency of early stimulating activities between the caregiver and child and caregiver knowledge of adequate dietary diversity are associated with improved developmental outcomes. Increased focus on supportive home environments and positive parenting practices may support children to reach their full cognitive and linguistic potential.

## Introduction

The early years of life are a crucial period for shaping an individual’s health, development, well-being and productivity. In the critical window from birth to 3 years of age, significant brain neuroplasticity amplifies both the impact of adverse threats to child development as well as the effects of interventions that promote optimal outcomes [1–3]. In many low- and middle-income country (LMIC) settings, limited early stimulation and learning opportunities, poor access to health care, and malnutrition and micronutrient deficiency contribute to a significant loss of potential [3, 4]. In 2015, global estimates suggested that over 250 million children under age 5 years (43%) were at risk of poor child development [5, 6].

Interventions aimed at improving early childhood development (ECD) in LMICs can have a significant impact, although timing and implementation characteristics are important components determining program success [7]. Reviews suggest that programs providing nutritional supplementation or education have variable and generally smaller impact on ECD outcomes [8]. Programs that promote caregiver stimulation or integrate stimulation with nutritional interventions have been more successful in improving ECD outcomes [9], although questions about optimal implementation at scale remain [10–12]. To make interventions impactful and sustainable, current recommendations emphasize implementing the Nurturing Care Framework as a multisectoral package, addressing health, nutrition, caregiver stimulation and early learning, and psycho-social threats to child development across the life course [13].

Sufficient dietary knowledge is one of the key factors that directly contributes to the nutrition provided to children, which in turn impacts a child’s development [14]. Further, stimulating caregiver child interactions are a critical proximal determinant of child development [15–18]. The process of caregivers engaging in activities such as book sharing, storytelling, singing songs, and counting or drawing with their children provides critical opportunities for early learning, which is directly linked to child development [19–22]. Among many other factors, frequency of early stimulation has been shown to be one of the greatest explanatory factors for the variability in ECD outcomes at a population level [23, 24]. These areas were selected for this analysis as they can be addressed at the community- and household-level without clinical intervention and are known to affect developmental outcomes in other contexts, but have yet to be explored in this context [11, 25].

There is little documentation of early parenting practices and ECD outcomes among families in Zanzibar, a semi-autonomous territory in the United Republic of Tanzania with a population of approximately 1.6 million including 275,000 children under age 5 [26]. Rates of chronic malnutrition, which are often used as a proxy measure for risk of poor ECD outcomes, indicate a moderately high level of risk with 21.5% of children in Zanzibar stunted [27]. We conducted a national household survey to describe parenting practices and ECD outcomes in Zanzibar. The primary goal of the survey was to serve as a baseline against which to measure the impact of a new national community health program on home environment, parenting parental knowledge and practices related to health, nutrition, and development, and ECD outcomes among children aged 18-29 months over time. In this paper, we present secondary analyses of the baseline cross-sectional survey that explore the associations between frequency of early stimulating activities between the caregiver and the child, dietary knowledge, and child development outcomes in Zanzibar, Tanzania.

## Methods

### Setting

In 2006, Zanzibar expanded access to early childhood education with a mandate for preschool education for all, though by 2015 still less than half of Zanzibar’s children had access to preschool [28]. Despite these initiatives to increase access to early learning, programs focusing on development during the critical window of the first 3 years of life remained unaddressed. In response, the Zanzibar Ministry of Health (MOH) and Ministry of Local Government (President’s Office Regional Administration and Local Government and Special Departments [PORALGSD]) launched the National Community Health Strategy 2019-2025, which formalized a cadre of over 2000 community health volunteers (CHVs) as part of the national health system with the creation of the *Jamii ni Afya* (‘Communities are Health’) program. The program aims to improve early childhood developmental outcomes by targeting promotion of, access to, and utilization of health care services, improved nutrition, and nurturing caregiver interactions for children in utero to age 5 and is built around WHO/UNICEF’s Nurturing Care Framework [13] and supported by a digital system co-developed with D-tree International.

### Sampling frame and participants

We conducted a cross-sectional, nationally-representative household survey in February 2019 in all 11 districts of Zanzibar, Tanzania. We used two-stage cluster sampling to randomly select 50 clusters using probability proportionate to size. Clusters and respective populations were defined by the enumeration areas provided by the Office of the Chief Government Statistician. We then used systematic random sampling within each cluster to identify and recruit 10 child-caregiver pairs from each cluster, for a total sample size of 500 participant pairs. Eligible participant pairs included children aged 18 to 29 months, with birth dates verified from their child health cards, and the child’s primary caregiver. Both caregiver and child were required to have their primary residence in Zanzibar. No other exclusion criteria were applied. Therefore, the sample is representative of the whole of Zanzibar, with all eligible children being equally likely to be selected for participation. Notably, this cross-sectional study takes place within a larger study to compare Caregiver Reported Early Developmental Index long-form (CREDI) scores at baseline to future survey implementations planned in 2023 and beyond. The power and sample size considerations for this larger study are provided in the supplemental materials (See Supplement 1).

### Data collection procedures

Household interviews with primary caregivers of eligible, enrolled children were conducted by trained data collectors in February 2019. The study questionnaire was approximately 40 min in length and administered in Kiswahili. The tool was previously translated and tested in Kiswahili in Tanzania by the tool’s developers. Data were collected on tablets and smartphones using ODK Collect. Data collectors and field supervisors were independent to the Ministry of Health and collaborating organizations, and participated in a 5-day training on ethical considerations, survey methodology, and administration of all data collection tools prior to data collection.

### Measures

The primary outcome measure was child development score as measured by the CREDI tool, which is based on caregiver report of easily observable and understandable child milestones and behaviors by age group. The tool has been validated in 17 low, middle- and high-income countries, including Tanzania [29, 30]. We report z-scores and raw scaled scores by language, cognitive, motor, and social-emotional domains, as z-scores are best used for comparison to other populations, and scaled scores are most appropriate for linear regression. For both measures, higher scores represent greater achievement in child development outcomes. Although the CREDI was not developed as a tool for diagnosing delay in individual children, we established a cut-off to define “developmental concern” for the purpose of comparison to the reference population and ease of communicating for policy and advocacy. We follow the conventions of other developmental assessment tools [31, 32] to consider to a z-score 1- < 2 standard deviations below the mean a “developmental concern” and “significant developmental concern” to be equal to a z-score 2 or more standard deviations below the mean. Because this is a standard normal distribution, we would expect 13.5% of children be in the “developmental concern” group. Likewise, we would expect 2.5% of children to be in the “significant developmental concern”. In accordance with CREDI guidelines, CREDI z-scores were used for descriptive analysis, while the continuous score was used for all hypothesis testing [33].

Our primary exposure variables of interest were caregiver reports of interactions with the child in the form of early stimulating activities and caregiver knowledge of dietary diversity. Questions were drawn from the UNICEF Multiple Indicator Cluster Survey (MICS) questionnaire. Caregiver report of interactions with the child was analyzed as a continuous variable defined as the summative number of early stimulating activities a child engaged in with any caregiver in the past 3 days. Types of early stimulating activities included: reading or looking at picture books, telling stories, singing songs, taking outside the home compound, playing a game, or naming/counting/drawing together. Knowledge of dietary diversity was the number of food groups the caregiver reported as appropriate for the child to eat, and knowledge of feeding frequency was the number times per day the caregiver reported the child should be fed. For the diet questions, we modified the MICS questions to reflect knowledge rather than practice, as the later was not feasible for implementation. The variables were categorized as those who named four or more food groups compared to those who named less, and those who named feeding thrice daily or more compared to those who suggested less. Knowledge of feeding practices were only assessed in a subgroup of children who were still breastfed and 18-23 months of age (*n* = 122).

We also gathered data on individual and household-level covariates related to the home environment, caregiver engagement and play, disciplinary practices, care-seeking behaviors, and health and nutritional knowledge and practices. All questions and indicators were defined and assessed using standard UNICEF indicators from MICS and a standardized monitoring and evaluation tool for UNICEF’s Care for Child Development checklist. We measured sociodemographic characteristics relevant to understanding the relationship between our independent exposures of interest and child development outcomes. Wealth was measured using the Tanzania EquityTool (https://www.equitytool.org/) which is a validated tool that analyzes household wealth using a simplified version of the DHS asset-based questionnaire. Using the EquityTool standard analysis package, each household was assigned a score and then categorized according to their relative wealth compared to quintile levels established by the Tanzania DHS 2015 population.

### Statistical analyses

We completed descriptive analyses of the overall and domain-specific CREDI z-scores and compared to the CREDI reference population^1^, using the CREDI scoring package developed in R V3.6.0 (R Core Team, Vienna, Austria). To explore associations between the individual-level covariates and overall and domain-specific CREDI continuous scores (herein: child development outcomes), we performed bivariate analyses for all categorical variables using Wald’s t-test and ANOVA. We fit two multivariate linear regressions to quantify the relationship between number of early stimulating activities and child development outcomes, and knowledge of adequate dietary diversity and child development outcomes. In both models, we adjusted for known confounding variables and those found to have a significant association in the bivariate analysis (at α = 0.05 significance level) including: geographic region, age of the caregiver, age and sex of the child, if caregiver is married or living with their partner or not, maternal and paternal education levels, parity, wealth, if the child was left alone for more than an hour in the past week, and (for caregiver engagement only) if the caregiver believed that domestic abuse was justifiable in any situation. We accounted for clustering by utilizing the svyset function on Stata. Our primary sampling unit was the enumeration area, and each individual was weighted by the probability of selection within their cluster. All tables and regression analyses account for the survey sampling plan our standard errors were adjusted accordingly. Given the difficulty to interpret meaningful changes in the raw scaled CREDI score, we standardized the results of our multivariate model analysis. To do so, we divided the coefficient of the CREDI outcome variable estimated by the model by the standard deviation within the study population for the specific CREDI domain, to express the effect size as change in standard deviation among the study population. With the exception of the CREDI scoring, all statistical analyses were performed using Stata Version 14 (StataCorp, College Station, TX).

### Ethical statement

Ethical approval to conduct this study was obtained from the institutional review boards at the Ministry of Health/Zanzibar Health Research Institute (Ref. No: ZAHREC/01/DEC/2018), and Boston Children’s Hospital (Ref. No.: P00029981). Every child’s parent or primary caregiver provided written informed consent on behalf of the child-caregiver pair prior to enrollment in the study, and all research was performed in accordance with approved study procedures and ethical guidelines.

## Results

### Participant characteristics

We enrolled 500 child-caregiver pairs. One pair was excluded from analysis as they were enrolled but did not have a child health card available. All 499 primary caregivers were women, a majority of whom were married and Muslim. For additional detail on the study population and characteristics of the home environment, refer to Tables 1 and 2, and Supplement 2.

**Table 1:** Participant characteristics

Variable	Total
	***n*** = 499
	n (%)
**Child age in months**	
18–<24 months	307 (61.5)
24–<30 months months	192 (38.5)
**Child sex**	
Male	253 (50.7)
Female	246 (49.3)
**Primary caregiver age group**	
18–24	82 (16.4)
25–29	131 (26.3)
30–34	116 (23.2)
35 or older	170 (34.1)
**Region**	
North Pemba	70 (14.0)
North Unguja	40 (80.2)
South Unguja	79 (15.8)
South Pemba	80 (16.0)
Urban West	230 (46.1)
**Tanzania National Wealth Index Quintile**	
Quintile 1 (Poorest)	28 (5.6)
Quintile 2	35 (7.0)
Quintile 3	63 (12.6)
Quintile 4	134 (26.9)
Quintile 5 (Wealthiest)	239 (47.9)
**Distance from Health Facility**	
Median	1.16 km
<2 km	427 (85.6)
2km or more	72 (14.4)
**Primary caregiver marital status**	
Single/Widowed	19 (3.8)
Married/Cohabited	456 (91.4)
Divorced	24 (4.8)
**Primary caregiver religion**	
Muslim	491 (98.4)
Christian	7 (1.4)
No religion	1 (0.2)
**Primary caregiver education completed**	
Primary School	104 (20.8)
Junior School	271 (54.3)
Senior, Vocational or Evening School	37 (7.4)
Diploma, Degree or Post-graduate Degree	14 (2.8)
Don’t know or no response	73 (14.6)
**Paternal education completed**	
Primary School	95 (19.0)
Junior School	242 (48.5)
Senior, Vocational or Evening School	52 (10.4)
Diploma, Degree or Post-graduate Degree	17 (3.4)
Don’t know or no response	93 (18.6)
**Primary caregiver parity**	
1–2	191 (38.28)
3–4	151 (30.26)
5 or greater	157 (31.46)
**Place of Delivery**	
Home	123 (24.6)
In transit/Other	25 (5.0)
Health Facility	351 (70.3)
**Danger Signs Primary Caregiver Can Recall**	
0–3	299 (59.9)
4 or more	200 (40.1)
**Abuse in the Home**	
Primary Caregiver Believes Domestic Abuse is Justifiable	143 (28.7)
Primary Caregiver Does Not Believe Domestic Abuse is Justifiable	356 (71.3)
**Physical Punishment**	
Used Physical Punishment	428 (85.8)
Did not use Physical Punishment	71 (14.2)
**Books in Home**	
No Books	446 (89.4)
One or More Books	53 (10.6)
**Child Left Alone**	
Left Alone for Over an Hour	79 (15.8)
Never Left Alone	420 (84.2)
**Maternal Engagement**	
Mother Did Not Engage in Activity with Child	32 (6.4)
Mother Engaged in at Least One Activity with Child	467 (93.6)
**Paternal Engagement**	
Father Did Not Engage in Activity with Child	256 (51.3)
Father Engaged in at Least One Activity with Child	243 (48.7)
**Food Diversity**	***n*** ** = 122**
Identifies Less than Four Food Groups Child Should be Eating	87 (71.3)
Identifies Four or More Food Groups Child Should be Eating	35 (28.7)
**Feeding Frequency Per Day**	***n*** ** = 122**
Less than 3	23 (18.9)
3 or more	98 (80.3)
Don’t know/Missing	1 (0.8)

**Table 2:** Participant characteristics and CREDI score, by domain

Variable	Overall Score Mean	SE	***P***-value	Motor score mean	SE	***P***-value	Cognitive score mean	SE	***P***-value	Linguistic score mean	SE	***P***-value	Social-Emotional score mean	SE	***P***-value
**Primary caregiver age group**			0.689			0.848			0.687			0.361			0.754
18–24	50.94	0.08		51.03	0.09		50.66	0.08		50.91	0.09		51.15	0.09	
25–29	50.97	0.07		51.10	0.10		50.68	0.07		50.92	0.08		51.16	0.06	
30–34	50.92	0.08		51.11	0.10		50.64	0.08		50.80	0.10		51.15	0.08	
35+	50.86	0.06		51.04	0.08		50.57	0.06		50.75	0.08		51.08	0.06	
**Region**			0.717			0.318			0.853			0.830			0.812
North Pemba	50.97	0.08		51.24	0.12		50.64	0.09		50.84	0.09		51.15	0.07	
North Unguja	50.83	0.09		50.96	0.09		50.53	0.10		50.77	0.10		51.06	0.10	
South Pemba	50.98	0.06		51.14	0.07		50.65	0.07		50.90	0.10		51.22	0.09	
South Unguja	50.90	0.12		50.91	0.19		50.68	0.10		50.94	0.14		51.09	0.13	
Urban West	50.90	0.05		51.05	0.06		50.64	0.05		50.81	0.07		51.12	0.05	
**National Wealth Index Quintile**			0.006			0.009			0.038			0.028			0.005
Quintile 1 (Poorest)	51.18	0.07		51.41	0.10		50.83	0.11		51.11	0.08		51.39	0.08	
Quintile 2	51.08	0.08		51.28	0.09		50.77	0.07		50.95	0.14		51.33	0.12	
Quintile 3	50.92	0.09		51.08	0.12		50.61	0.10		50.90	0.10		51.08	0.09	
Quintile 4	50.85	0.07		51.00	0.07		50.57	0.07		50.76	0.08		51.08	0.08	
Quintile 5 (Wealthiest)	50.89	0.05		51.04	0.07		50.63	0.05		50.81	0.06		51.11	0.05	
**Distance from Home to Health Facility**			0.844			0.165			0.459			0.585			0.149
< 2 km	50.90	0.08		51.23	0.12		50.58	0.07		50.78	0.10		51.02	0.08	
2 km or more	50.92	0.04		51.04	0.05		50.64	0.04		50.84	0.05		51.15	0.04	
**Primary caregiver marital status**			0.350			0.233			0.263			0.994			0.248
Single/Widowed/Divorced/Separated	50.80	0.13		50.90	0.16		50.50	0.13		50.83	0.15		50.98	0.14	
Married/Living with Partner	50.93	0.03		51.09	0.04		50.64	0.03		50.83	0.04		51.14	0.03	
**Primary caregiver education completed**			0.302			0.175			0.528			0.417			0.476
Primary School	50.99	0.08		51.20	0.10		50.68	0.08		50.92	0.10		51.17	0.07	
Junior School	50.84	0.05		50.96	0.06		50.58	0.05		50.77	0.06		51.06	0.05	
Senior, Vocational or Evening School	51.01	0.12		51.15	0.18		50.71	0.11		50.96	0.15		51.20	0.11	
Diploma, Degree or Post-graduate Degree	50.95	0.20		51.17	0.25		50.71	0.19		50.76	0.24		51.19	0.20	
**Paternal Education Completed**			0.044			0.032			0.104			0.065			0.064
Primary School	50.88	0.07		51.10	0.09		50.58	0.07		50.73	0.09		51.11	0.07	
Junior School	50.87	0.05		50.99	0.05		50.60	0.05		50.81	0.06		51.08	0.04	
Senior, Vocational or Evening School	51.16	0.10		51.41	0.13		50.83	0.09		51.10	0.12		51.31	0.10	
Diploma, Degree or Post-graduate Degree	51.07	0.16		51.24	0.22		50.79	0.15		50.86	0.20		51.40	0.15	
**Parity**			0.003			0.009			0.001			0.030			0.013
1–2	50.77	0.06		50.91	0.07		50.47	0.06		50.70	0.07		50.99	0.05	
3–4	51.00	0.07		51.15	0.08		50.73	0.07		50.91	0.08		51.22	0.07	
≥ 5	51.01	0.05		51.19	0.07		50.74	0.05		50.92	0.06		51.21	0.05	
**Sex of Child**			0.911			0.967			0.899			0.889			0.904
Male	50.92	0.05		51.07	0.06		50.64	0.05		50.84	0.06		51.13	0.05	
Female	50.91	0.06		51.07	0.08		50.63	0.06		50.83	0.07		51.12	0.05	
**Child’s Delivery Location**			0.273			0.511			0.358			0.175			0.279
Home/Community Delivery	50.94	0.04		51.09	0.05		50.65	0.04		50.86	0.05		51.15	0.04	
Health Facility Delivery	50.86	0.05		51.02	0.08		50.59	0.05		50.76	0.06		51.08	0.06	
**Danger Signs Primary Caregiver Can Recall**			0.371			0.473			0.519			0.376			0.282
0–3	50.94	0.05		51.09	0.06		50.65	0.05		50.87	0.06		51.16	0.05	
4+	50.87	0.05		51.03	0.06		50.60	0.05		50.78	0.07		51.08	0.04	
**Abuse in the Home**			0.813			0.957			0.786			0.758			
Primary Caregiver Believes Domestic Abuse is Justifiable	50.90	0.05		51.07	0.07		50.62	0.05		50.81	0.06		51.11	0.05	
Primary Caregiver Does Not Believe Domestic Abuse is Justifiable	50.92	0.04		51.07	0.05		50.64	0.04		50.84	0.05		51.13	0.04	
**Physical Punishment**			0.152			0.847			0.075			0.057			0.127
Used Physical Punishment	50.94	0.04		51.07	0.05		50.66	0.04		50.86	0.05		51.15	0.04	
Did not use Physical Punishment	50.79	0.09		51.05	0.13		50.47	0.09		50.64	0.10		50.98	0.10	
**Books in Home**			0.790			0.483			0.989			0.753			0.792
No Books	50.94	0.10		51.15	0.12		50.63	0.10		50.86	0.12		51.10	0.10	
One or More Books	50.91	0.03		51.06	0.05		50.63	0.03		50.83	0.04		51.13	0.03	
**Child Left Alone**			0.835			0.558			0.649			0.761			0.520
Left Alone for Over an Hour	50.90	0.09		51.13	0.10		50.59	0.09		50.80	0.11		51.07	0.09	
Never Left Alone	50.92	0.04		51.06	0.05		50.64	0.04		50.84	0.05		51.14	0.04	
**Maternal Interactions**			0.217			0.043			0.508			0.327			0.601
Mother Did Not Engage in Any Early Stimulation Activity with Child	50.72	0.16		50.67	0.21		50.53	0.15		50.64	0.20		51.05	0.17	
Mother Engaged in at Least One Early Stimulation Activity with Child	50.93	0.03		51.10	0.04		50.64	0.04		50.84	0.04		51.13	0.03	
**Paternal Interactions**			0.585			0.218			0.727			0.946			0.747
Father Did Not Engage in Any Early Stimulation Activity with Child	50.90	0.05		51.02	0.06		50.62	0.05		50.83	0.07		51.12	0.05	
Father Engaged in at Least One Early Stimulation Activity with Child	50.94	0.05		51.12	0.06		50.64	0.05		50.83	0.05		51.14	0.05	
**Food Diversity (** ***n*** **= 122)**			0.004			0.005			0.006			0.006			0.032
Identifies Less than Four Food Groups Child Should be Eating	50.52	0.08		50.64	0.10		50.35	0.07		50.31	0.10		50.77	0.07	
Identifies Four or More Food Groups Child Should be Eating	50.97	0.12		51.17	0.14		50.76	0.12		50.85	0.15		51.11	0.12	
**Feeding Frequency (** ***n*** **= 122)**			0.266			0.311			0.321			0.348			0.260
Less than 3	50.49	0.14		50.61	0.17		50.34	0.13		50.31	0.19		50.72	0.14	
3 or more	50.69	0.08		50.85	0.11		50.50	0.07		50.51	0.09		50.92	0.07	

### Child development outcomes

**Fig. 1:**
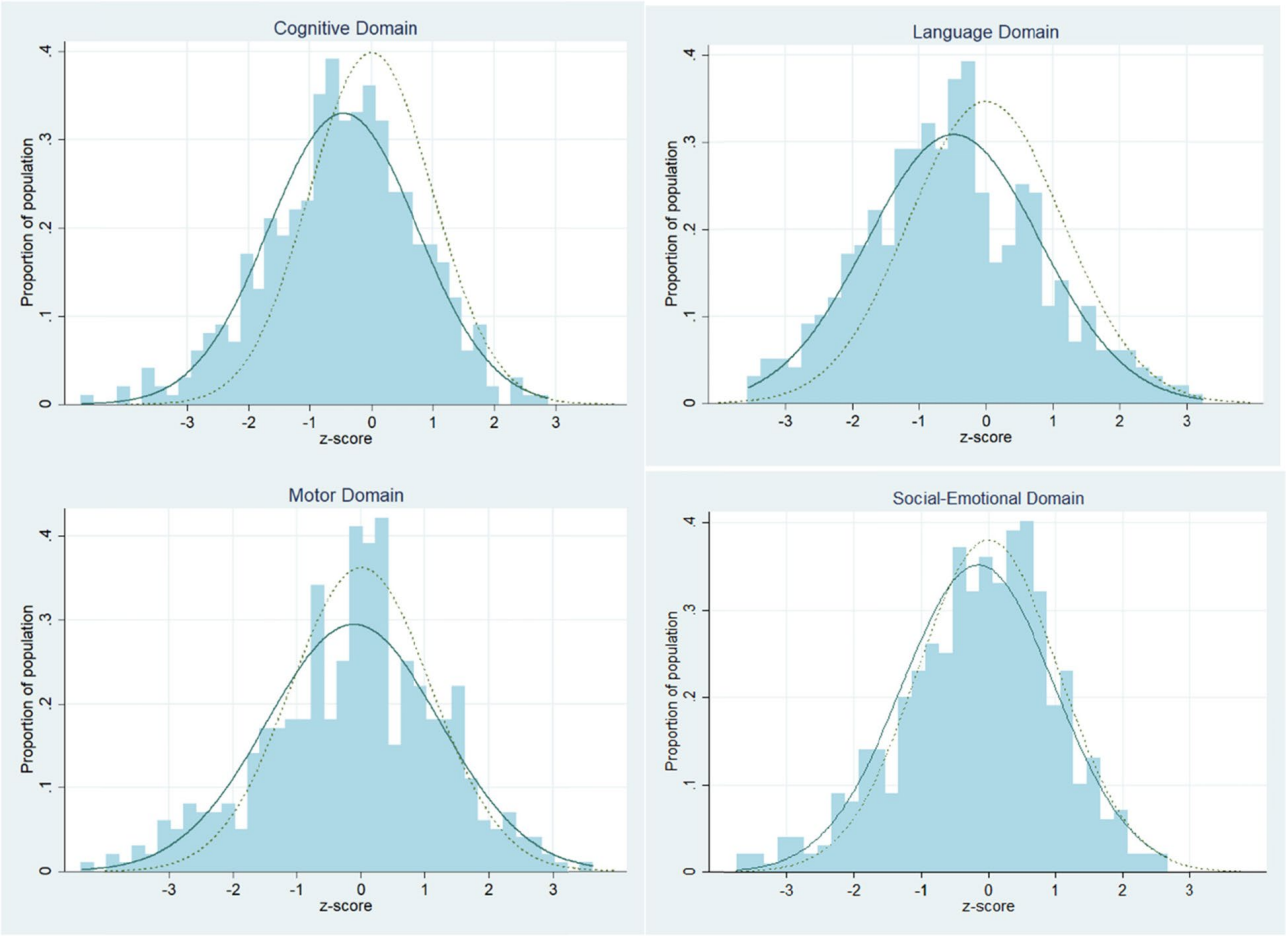
Distribution of CREDI scores among sampled population (solid) compared to reference population (dotted)

In Zanzibar, 9.6% of sampled children, that is, more than 3 times the proportion of children in the reference population, fell into the area of significant developmental concern (i.e., z-score greater than or equal to 2 SD below the reference mean) for the overall CREDI developmental score. An additional 18.4% had CREDI z-scores between one and two SD below the reference mean. Mean z-scores ranged from − 0.116 (SD: 1.354, range: − 4.372 to 3.449) in the motor domain, to − 0.494 (SD: 1.309, range: − 5.275 to 3.199) in the language domain (Table 3). The left shift of the normal distribution of the development curve signifies that a greater number of young children in Zanzibar have developmental difficulties compared to the reference population (Fig. 1). The poorest performing domains were language and cognitive with 12.7% and 10.2% of children within area of significant concern, respectively. The motor domain had 9.6% of children within the area of significant concern, followed by social-emotional domain with 6.2% of children.

**Table 3:** Early childhood development CREDI z-scores compared to reference population, by domain

CREDI z-score (***n*** = 499)	Mean	SD	Min	Max
Cognitive	−0.457	1.208	−4.725	2.716
Language	−0.494	1.309	−5.275	3.199
Motor	−0.116	1.354	−4.372	3.449
Social-emotional	− 0.151	1.130	−3.730	2.641

### Parenting practices

**Fig. 2:**
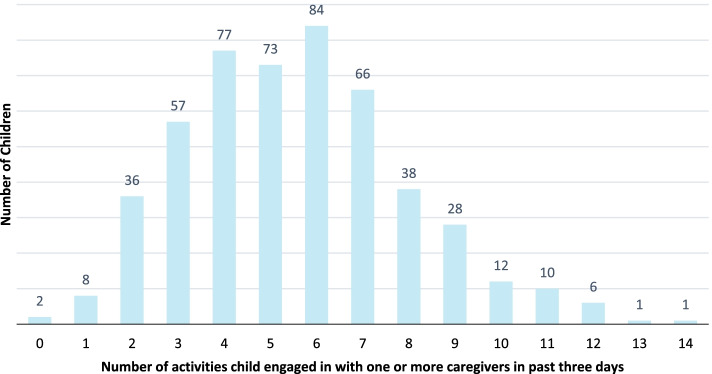
Distribution of number of early stimulating activities children engaged in in previous 3 days with any caregiver or household member over the age of 15

One-fifth (20.6%) of children engaged in 0-3 activities with their caregivers in the past 3 days, 30.1% engaged in 4-5 activities, 30.1% engaged in 6-7 activities, and 19.2% engaged in over 8 activities (Fig. 2). The most common activities were taking the child outside the home or compound and playing, while the least common were telling stories and reading or looking at books (Table 4). Maternal interaction with their children was more frequent than paternal, with 94% of mothers playing with their child at least once in the previous 3 days, compared to only 49% of fathers. On average, mothers engaged in 2.7 (SD: 1.4) different types of early stimulating activities with their children in the past 3 days, compared to fathers who engaged in only 0.8 types (SD: 1.0). The majority of caregivers (89%) reported having no children’s books at home. Half of children (51%) played with toys made at home, and the majority of children (75%) also played with common household objects such as bowls or pots, or objects found outside, such as sticks, rocks, animal shells or leaves. Children engaged in a median time of 3 hours of play (IQR: 1-7 h) in total with all caregivers and household members age 15 or older in the previous 3 days.

**Table 4:** Early stimulation activities with children by care provider in past 3 days

Activity (*N *= 499)	Mother	Father	Others	None	Don’t know
	n (%)	n (%)	n (%)	n (%)	n (%)
Read books to or looked at picture books	68 (13.6)	23 (4.6)	101 (20.2)	340 (68.1)	9 (1.8)
Told stories	61 (12.2)	19 (3.8)	72 (14.4)	367 (73.6)	10 (2.0)
Sang songs, including lullaby	281 (56.3)	43 (8.6)	181 (36.3)	132 (26.5)	1 (0.2)
Took child outside the home, compound, yard or enclosure	336 (67.3)	130 (26.1)	233 (46.7)	42 (8.4)	4 (0.8)
Played	340 (68.1)	120 (24.1)	281 (56.3)	53 (10.6)	1 (0.2)
Named, counted, or drew things	252 (50.5)	66 (13.2)	168 (33.7)	169 (33.9)	6 (1.2)

Among caregivers with children under 2 years of age and still breastfeeding, 28.7% of caregivers could name four or more food groups the child should be eating, and 80.3% said their child should be fed solid foods three or more times per day.

### Association between frequency of early stimulating activities and child development outcomes

In our multivariate model, an increased number of early stimulating activities was associated with improved child development outcomes across all CREDI domains (adjusted *p*-values: 0.002 to 0.017) (Table 5). Overall, in the adjusted model, each additional activity any caregiver does with their child was associated with a 0.036 point increase in overall CREDI score (95% CI = (0.014, 0.058), *p* = 0.002). The strongest associations were seen in the motor and language domains, where one additional activity was associated with a 0.039 point increase (95% CI = (0.011, 0.066), *p* = 0.007) in motor development and 0.039 point increase (95% CI = (0.007, 0.053), *p* = 0.017) in language development CREDI scores in the adjusted model. The weaker associations were seen in the cognitive and social-emotional domains, with one additional activity associated with 0.037 point (95% CI = (0.014, 0.059), *p* = 0.002) and 0.030 point (95% CI = (0.007, 0.053), *p* = 0.010) increases in CREDI score in the adjusted model, respectively. Standardizing the results, we found engaging on average in one more activity during the three-day recall period translated to 0.094 SD higher score on the cognitive domain, 0.135 SD higher score on the motor domain, 0.117 SD higher score on the language domain and 0.105 SD higher score in the social emotional domain.

**Table 5:** Caregiver interactions (number of early stimulation activities) and CREDI Outcome by Domain

	Unadjusted			Adjusted^a^		
CREDI Domain	Beta Coefficient	95% CI	***p***-value	Beta Coefficient	95% CI	***p***-value
**Overall Score**	0.034	(0.010, 0.059)	0.008	0.036	(0.014, 0.058)	0.002
**Cognitive**	0.032	(0.007, 0.056)	0.012	0.037	(0.014, 0.059)	0.002
**Language**	0.035	(0.003, 0.067)	0.031	0.039	(0.007, 0.072)	0.017
**Social-Emotional**	0.030	(0.004, 0.057)	0.025	0.030	(0.007, 0.053)	0.010
**Motor**	0.039	(0.010, 0.069)	0.009	0.039	(0.011, 0.066)	0.007

^a^Adjusted for region, primary caregiver age, child’s age, sex, marital status of caregiver, maternal and paternal educational status, parity, wealth quintile, child left alone, and tolerance for violence in home

### Association between dietary diversity knowledge and child development outcomes

Children with caregivers that could name four or more food groups to feed their child scored 0.564 points higher in their overall CREDI development score than those who named fewer than four (95% CI = (0.281, 0.846), *p* < 0.001) (Table 6). Greater knowledge of adequate dietary diversity was associated with higher CREDI scores across all four domains in both unadjusted and adjusted models. The association was strongest in the language and motor domains, with children whose caregivers named at least four food groups scoring on average 0.677 point (95% CI = (0.365, 0.988), *p* < 0.001) and 0.592 point (95% CI = (0.229, 0.956), *p* = 0.002) higher even after adjusting for household wealth. Children with caregivers with adequate knowledge of food diversity had a 0.710 SD higher score in the motor domain, a 0.694 SD higher score in the language domain, a 0.541 SD higher score in the social emotional domain, and a 0.530 SD higher score in the cognitive domain. When included in the final multivariate model, knowledge of minimum feeding frequency was not associated with development outcomes in any domain and thus excluded from the model. Knowledge of dietary diversity was not associated with a family’s wealth, with approximately 29% of caregivers across all wealth quintiles able to name four or more food groups to feed their children regularly.

**Table 6:** Knowledge of dietary diversity (four or more food groups compared to fewer than four) and CREDI outcome by domain

CREDI Domain	Unadjusted			Adjusted^a^		
	Beta Coeff.	95% CI	***p***-value	Beta Coeff.	95% CI	***p***-value
**Overall Score**	0.455	(0.152, 0.758)	0.004	0.564	(0.281, 0.846)	< 0.001
**Cognitive**	0.412	(0.122, 0.701)	0.006	0.547	(0.265, 0.828)	< 0.001
**Language**	0.532	(0.158, 0.906)	0.006	0.677	(0.365, 0.988)	< 0.001
**Social-Emotional**	0.342	(0.032, 0.652)	0.032	0.439	(0.124, 0.754)	0.007
**Motor**	0.535	(0.166, 0.904)	0.005	0.592	(0.229, 0.956)	0.002

^a^Adjusted for region, primary caregiver age, child’s age, sex, marital status of caregiver, maternal and paternal educational status, parity, wealth quintile, and child left alone

## Discussion

Overall, Zanzibari children aged 18-29 months scored markedly lower in motor, linguistic, cognitive, and socio-emotional domains than the global reference population, with social emotional domains a relative area of strength. In total, 28% of Zanzibari children fell in the area of developmental concern, compared to 17.5% in the reference population. The Zanzibari population had higher average CREDI scores compared to other findings of early developmental status in Mainland Tanzania [34], which is consistent with Zanzibar’s relatively high Human Development Index (HDI) (regional range: 0.543 to 0.690) compared to Tanzania overall (national HDI score: 0.529; regional range: 0.464 to 0.690 including Zanzibar) [35].

While our study design limits our ability to attribute causation, we found a strong association between caregiver interactions, in the form of regular play and communication and early stimulation activities, and child development outcomes. This association is evident across all developmental domains, is dose-dependent on number of caregiver child activities and incremental increases in developmental outcomes, and strengthened when we adjusted for wealth, parental education, and geography. This finding is consistent with other ECD studies, which show the positive impact of coaching of caregiver stimulation on developmental outcomes [23, 24, 36], and strong positive associations between caregiver engagement, child stimulation, and higher CREDI scores. Our findings highlight the importance of testing the impact of interventions that incorporate strong and effective approaches to coach parents and caregivers to engage in more play and communication activities with their children on ECD outcomes, as this could contribute to an overall increase in CREDI scores in this population. However, while increases in parental engagement activity were significantly linked to developmental outcomes, the magnitude of this association was moderate, and suggests that other factors are also contributing to the variation in ECD outcomes, and therefore that complementary health and nutrition interventions should be linked to parent stimulation intervention to promote optimal early childhood development.

Our analysis also revealed an association between a parent’s knowledge of adequate dietary diversity and development outcomes, which was significant after controlling for wealth, child’s age, and parental education. Associations between nutritional deficiencies, including stunting and micronutrient deficiency and developmental outcomes are well documented [37]. Previous studies have also indicated the importance of nutrition education interventions, in addition to interventions targeting food security alone, in improving dietary diversity and children’s growth [38, 39]. As we only assessed parents’ knowledge rather than nutritional practices, the mechanism underlying the association between nutritional knowledge and developmental outcome will need to be elucidated in future research. Given the positive association identified even after controlling for wealth, and the known link in literature between knowledge, dietary diversity, and child growth, there is an opportunity to further explore the benefit of household nutrition education provided by CHVs in Zanzibar on child growth and development through future studies.

This study is the first known report of early developmental outcomes at the population level in Zanzibar. While many reports of developmental status in LMIC settings rely on proxy measures such as poverty or stunting, or utilize more complex measures (e.g., Bailey, MDAT) on a subset of the population, our use of the CREDI, an internationally-validated simple caregiver reporting tool in a nationally representative survey provides a unique first assessment of ECD status across Zanzibar. Our findings are novel to the Zanzibar context and can be used, along with best practices from the literature, to serve as an important baseline for evaluating the success of subsequent programs, developing and testing interventions to promote optimal child development in resource-constrained settings, and contributing to the limited ECD literature in this context.

There were some limitations to our data collection approach, which limited the analyses we could conduct and conclusions we could draw. The primary limitation is the cross-sectional nature of the study, which allows us to identify associations but not draw causal inference. In subsequent planned studies part of the *Jamii ni Afya* program, we will be able to elucidate causal influences using longitudinal, individual-level data collected. Other limitations are that we were not able to assess quality or duration of each play activity, and that we use the past 3 days of caregiver engagement as a proxy for usual engagement. Despite these limitations, our decision to utilize questions from UNICEF’s MICS and the CREDI tool provides the opportunity to compare our results to other countries who have utilized the same survey instruments.

With the support of a digital system that integrates client tracking, decision support, coaching and counseling, and audio/visual messages, Zanzibar’s CHVs will be able to deliver integrated ECD, health, and nutrition services. As the Ministry of Health and D-tree International jointly implement and scale the *Jamii ni Afya* program nationwide in Zanzibar, prospective data collection on a range of household and individual factors which affect child development and growth will provide a rich opportunity to rigorously explore these initial findings, overcoming some of the limitations of this analysis, and test the effectiveness of various interventions in improving children’s health, development, and overall well-being.

## Conclusion

Our findings show a positive association between quantity of early stimulating activities and ECD outcomes, as well as caregiver knowledge of adequate dietary diversity and ECD outcomes. In combination with existing supporting evidence, these findings provide a direction for developing and testing parenting interventions at the household and community-levels in the Zanzibari population, in combination with other health and nutrition interventions to improve child development.

## Supplementary Information

**Additional file 1.** Sample size calculation for baseline and endline household survey.

**Additional file 2.** Additional Participant Characteristics, Analysis, and Interpretation.

## Data Availability

The datasets used and/or analyzed during the current study are available from the corresponding author on reasonable request.

## Abbreviations

ANOVA: Analysis of Variance
DHS: Demographic and Health Survey
ECD: Early Childhood Development
CHV: Community Health Volunteer
CREDI: Caregiver-reported Development Instrument – Long-form
MOH: Ministry of Health
PORALGSD: President’s Office Regional Administration and Local Government and Special Departments
UNICEF: United Nations Children’s Fund

## Footnotes

1 The CREDI reference population comprises all children in the original CREDI data base with an “ideal” home environment. Ideal home environments were defined through maternal educational attainment (college or higher), as well as through the number of activities done by adults with the child in the last 3 days (at least 4 out of the 6 MICS home stimulation activities). (CREDI Scoring Manual)
