# Supplementary material for: Caregiver parenting practices, dietary diversity knowledge, and association with early childhood development outcomes among children aged 18-29 months in Zanzibar, Tanzania: a cross-sectional survey
Source: BMC Public Health. 2022 Apr 15;22:762. doi: 10.1186/s12889-022-13009-y (PMC9012040; doi:10.1186/s12889-022-13009-y)
Supplement: Supplementary file 1 — Additional file 1. Sample size calculation for baseline and endline household survey. [file 12889_2022_13009_MOESM1_ESM.docx]

**Title:**

Caregiver parenting practices, dietary diversity knowledge, and association with early childhood development outcomes among children aged 18-29 months in Zanzibar, Tanzania: A cross-sectional survey

Supplement 1: Sample size calculation for baseline and endline household survey

The data used for this secondary analysis were drawn from the baseline household survey for the Jamii ni Afya program. The same sampling methodology described will be used to recruit an additional, independent sample of 500 child-caregiver pairs at endline in 2023. The sample size needed for the baseline and endline measurement comparison was calculated to detect any 0.5-point change in the child development cognitive domain score, as measured by the Caregiver-Reported Early Development Index (CREDI), with type I error of 5% and power of 80%, assuming a standard deviation of 1.4 points. We applied a design effect of 2.0 to account for increased variance due to cluster sampling, unknown variance in the outcome indicator in our study population, and to account for non-response. Our target sample size was 250 respondents in each age group defined by CREDI, 18-23 months and 24-29 months, at each time point. While this sample size was calculated for the purpose of the baseline and endline survey, we present it here to demonstrate the rationale for our sample size, and to show the degree of precision attainable in the baseline measurement.
