# Supplementary material for: Caregiver parenting practices, dietary diversity knowledge, and association with early childhood development outcomes among children aged 18-29 months in Zanzibar, Tanzania: a cross-sectional survey
Source: BMC Public Health. 2022 Apr 15;22:762. doi: 10.1186/s12889-022-13009-y (PMC9012040; doi:10.1186/s12889-022-13009-y)
Supplement: Supplementary file 2 — Additional file 2. Additional Participant Characteristics, Analysis, and Interpretation. [file 12889_2022_13009_MOESM2_ESM.docx]

**Supplement 2: Additional Participant Characteristics, Analysis, and Interpretation**

Approximately 85% of both caregivers and their partners had ever attended school, with only 10% having completed secondary school or a higher level of education. The average age of enrolled children was 22.7 months, and half of enrolled children (n=253, 50.7%) were male. In our study population, 85.8% of caregivers reported engaging in some form of physical punishment with their child in the past month. Only 5.8% of caregivers reported engaging in only non-violent discipline (e.g., explaining or withdrawing privileges) (Supplemental Figure 1). Two-thirds of caregivers engaged in both physically and psychologically aggressive (such as shouting/yelling at child, or calling him/her names) disciplinary methods (Table 1).

Prior research has shown that specific aspects of the home environment and caregiving practices can adversely impact ECD outcomes.^S1^ There were several additional findings that require elucidation in future studies with longitudinal data. First, we found that children with higher maternal parity were more likely to have higher development scores (p=0.003 for overall CREDI score) (Table 2). The literature describes a strong link between young maternal age, presumably linked to lower parity, and poorer child health and development outcomes.^S2^ Differences in maternal interactions are reported to vary between first time and more experienced mothers, with multiparous mothers having increased self-reported maternal parenting efficacy and sensitivity.^S2-S5^ Second, while wealth was associated with the child development outcomes in the bivariate analysis, this association was not significant in the multivariate model. We believe this could be because three-quarters of sampled Zanzibari households were in the top two Tanzania national wealth quintiles, and therefore there was not enough variation among the sample to observe the association with development outcomes. Similarly, we did not see an association between maternal or paternal education and child development outcomes after adjusting for other factors. This could be because only 12% of mothers and 17% of fathers completed senior high school or higher levels of education, where we would expect to see improved child development outcomes. Third, much literature on the science of early brain development documents the impact of toxic stress from exposure to violence on childhood well-being^S3^, as well as the impact of parent stimulation on mitigating the impact of violence on developmental outcomes.^S6^ We found high levels of physical and harsh verbal discipline and acceptance of use of interpersonal violence in the home, all of which are known to adversely impact a child’s development.^S3^ We did not find an association between report of acceptance of physical violence in the home or report of physical and harsh verbal discipline on ECD outcomes. While we utilized standard MICS questions of endorsement of interpersonal violence, the question asks attitudes toward violence rather than home experience so may lack sensitivity in measuring a child’s true exposure to violence. As only 5.8% of caregivers reported engaging in only positive disciplinary practices, our analyses lacked sufficient power to quantify the differences in child development between households that reported physical or harsh punishment, positive punishment, or no punishment at all.

**Supplemental Figure 1: Disciplinary and punishment methods used by adults in the household with the child in the previous month**

**References: Supplement 2**

S1. Yousafzai A, Rasheed M, Rizvi A, Robert Armstrong R, Bhutta ZA. Effect of integrated responsive stimulation and nutrition interventions in the Lady Health Worker programme in Pakistan on child development, growth, and health outcomes: a cluster-randomised factorial effectiveness trial. Lancet. 2014 Oct;384(9950):1282-93.

S2. Fall CH, Sachdev HS, Osmond C, Restrepo-Mendez MC, Victora C, Martorell R, Stein AD, Sinha S, Tandon N, Adair L, Bas I, Norris S, Richter LM; COHORTS investigators. Association between maternal age at childbirth and child and adult outcomes in the offspring: a prospective study in five low-income and middle-income countries (COHORTS collaboration). Lancet Glob Health. 2015 Jul;3(7):e366-77.

S3. Thompson, R. Stress and Child Development. The Future of Children. 2014; 24(1), 41-59. Accessed December 12, 2020, from <http://www.jstor.org/stable/23723382>.

S4. Falster K, Hanly M, Banks E, Lynch J, Chambers G, Brownell M, et al. Maternal age and offspring developmental vulnerability at age five: A population-based cohort study of Australian children. PLoS Med. 2018; 15(4): e1002558.

S5. Fish M and Shifter T. Mother parity as a main and moderating influence on early mother-infant interaction. Journal of Applied Developmental Psychology. 1993;14(4):557-572.

S6. Jeong J, McCoy DC, Yousafzai AK, Salhi C, Fink G. Paternal Stimulation and Early Child Development in Low- and Middle-Income Countries. Pediatrics. 2016 Oct;138(4):e20161357.
